# Production of erythritol and mannitol by *Yarrowia lipolytica* yeast in media containing glycerol

**DOI:** 10.1007/s10295-012-1145-6

**Published:** 2012-05-31

**Authors:** Ludwika Tomaszewska, Anita Rywińska, Witold Gładkowski

**Affiliations:** 1 Department of Biotechnology and Food Microbiology, Wrocław University of Environmental and Life Sciences, Chełmońskiego 37/41, 51-630 Wrocław, Poland; 2 Department of Chemistry, Wrocław University of Environmental and Life Sciences, Norwida 25/27, 50-375 Wrocław, Poland

**Keywords:** *Yarrowia lipolytica*, Glycerol, Erythritol, Mannitol, Arabitol

## Abstract

Glycerol is a by-product generated in large amounts during the production of biofuels. This study presents an alternative means of crude glycerol valorization through the production of erythritol and mannitol. In a shake-flasks experiment in a buffered medium, nine *Yarrowia lipolytica* strains were examined for polyols production. Three strains (A UV’1, A-15 and Wratislavia K1) were selected as promising producers of erythritol or/and mannitol and used in bioreactor batch cultures and fed-batch mode. Pure and biodiesel-derived crude glycerol media both supplemented (to 2.5 and 3.25 %) and not-supplemented with NaCl were applied. The best results for erythritol biosynthesis were achieved in medium with crude glycerol supplemented with 2.5 % NaCl. Wratislavia K1 strain produced up to 80.0 g l^−1^ erythritol with 0.49 g g^−1^ yield and productivity of 1.0 g l^−1^ h^−1^. Erythritol biosynthesis by A UV’1 and A-15 strains was accompanied by the simultaneous production of mannitol (up to 27.6 g l^−1^). Extracellular as well as intracellular erythritol and mannitol ratios depended on the glycerol used and the presence of NaCl in the medium. The results from this study indicate that NaCl addition to the medium improves erythritol biosynthesis, and simultaneously inhibits mannitol formation.

## Introduction

Glycerol is generated in large amounts by the biofuel industry during the production of bioethanol [1] and biodiesel [46]. Its use as a medium component in microbiological synthesis for the production of valuable products has been discussed widely in the last decade. Many microorganisms are known to utilize glycerol naturally as a sole carbon and energy source in both aerobic and anaerobic processes. Although the fermentative metabolism of glycerol has been studied in great detail in several species of the *Enterobacteriaceae* family [51], the number of studies dealing with the utilization of glycerol by moulds or yeast is still limited.

It is known that in the case of *Yarrowia lipolytica* yeast, glycerol acts very well as a carbon source along with carbohydrates, *n*-alkanes, fatty acids and oils. In medium containing both glycerol and glucose, the yeast first utilizes glycerol [32, 40]. *Y. lipolytica* yeast are distinguished by their unusual physiological and biological properties, which makes this genus play an important role in biosynthesis as well as in biodegradation and biotransformation processes [5, 15]. *Y. lipolytica* are able to produce a wide spectrum of organic acids from the TCA cycle such as citric, isocitric, pyruvic and α-ketoglutaric from various substrates, including glycerol [9]. An alternative method of glycerol valorisation involves its biotransformation to a single cell oil (SCO) by oleaginous moulds or yeast like *Y. lipolytica* [31]. Particular attention was paid to the biosynthesis of citric acid. The effects of different kinds of raw glycerol on yeast growth and kinetic parameters of citric acid production in batch, fed-batch and continuous culture were described [39–41]. The selectivity of citric acid biosynthesis, including the share of isocitric, malic, ketoglutaric acids and polyols, has also been studied [39, 40]. The results of these studies showed that erythritol and mannitol were also produced during the production of citric acid from glycerol by *Y. lipolytica* [36, 40]. In addition, a large amount of erythritol was produced by a selected mutant of *Y. lipolytica* (Wratislavia K1) on a glycerol-based medium at low pH [37].

Erythritol is a four-carbon sugar alcohol produced in microbiological processes. In comparison to other sugar alcohols used currently as sucrose replacers, erythritol has a much lower energy value (~0.2 kcal g^−1^) than sucrose (4 kcal g^−1^) and other sugar alcohols (~2.4 kcal g^−1^). Moreover, this polyol is non-cariogenic, generally free of gastric side-effects in regular use and its use in food is largely approved [27, 35].

Mannitol—a six-carbon sugar alcohol—has several applications in the food, pharmaceutical and medical industries. It is distributed widely in nature, being found in various plants, algae, in the mycelia of various fungi and it is one of the main carbohydrates in mushrooms. It is currently produced industrially by chemical synthesis using hydrogenation of fructose at high temperature and pressure. This process is not very efficient and requires a high purity of substrates. Therefore, microbiological production of mannitol would be an interesting alternative [42, 45].

It is known that osmophilic yeast-like fungi and some bacteria can produce sugar alcohols or their derivatives in response to increased external osmotic pressure [13, 49]. Most yeast produce trace amounts of one or more sugar alcohols, but more effective biosynthesis have been obtained by species that display a relatively high tolerance for high concentrations of salts or sugars [26]. Since *Y. lipolytica* yeast is capable of growing even in the presence of 12 % NaCl in the medium [3], the possible use of this yeast as a producer of polyols may be of great interest.

The aim of this study was to examine the ability of *Y. lipolytica* to produce erythritol and mannitol from glycerol both in the presence and absence of sodium chloride.

## Materials and methods

### Microorganisms

Strains CCY-29-26-3 and CCY-29-26-4 of *Y. lipolytica* were from the Czechoslovak Collection of Yeasts. The wild strains A-1, A-2-4, A-3, A-6, A-15, the UV-mutant A UV’1, and the acetate-negative mutants Wratislavia 1.31 and Wratislavia K1 of *Y. lipolytica* used in this study came from the collection belonging to the Department of Biotechnology and Food Microbiology at Wrocław University of Environmental and Life Sciences, Wrocław, Poland. The mutants were isolated from wild strains after their exposure to UV radiation: UV’1 from parental ATCC 8661 and Wratislavia 1.31 from A-101 strain. *Y. lipolytica* Wratislavia K1 strain was isolated from strain Wratislavia 1.31 in the course of continuous citric acid production from glucose in a nitrogen-limited chemostat culture at a dilution rate of* D* = 0.016 h^−1^. The yeast strains were maintained on YM slants at 4 °C.

### Substrates

Pure glycerol 98 % wt wt^−1^ (POCH, Gliwice, Poland) or crude glycerol derived from biodiesel production (Wratislavia-BIO, Wrocław, Poland) containing 87.3 % wt wt^−1^ glycerol and 4.3 % wt wt^−1^ NaCl were used as carbon and energy sources.

### Media

The growth medium contained: pure glycerol 50.0 g; yeast extract 3.0 g; malt extract 3.0 g; bactopeptone 5.0 g; and distilled water to 1 l. The medium for the shake-flasks experiment consisted of: pure glycerol 100 g; NH_4_Cl 4.56 g; MgSO_4_ · 7H_2_O 1 g; yeast extract 1 g; CuSO_4_ 0.7 × 10^−3^ g; MnSO_4_ · H_2_O 32.6 × 10^−3^ g; and 0.72 M phosphate buffer pH 4.3 to 1 l. Production media in 1 l tap water contained: carbon source 150 g (batch cultures) or 250 g (fed-batch cultures); NH_4_Cl 2.0 g; KH_2_PO_4_ 0.2 g; MgSO_4_ · 7H_2_O 1.0; yeast extract 1.0 g; NaCl 25, 32.5 g or none. To obtain 150 g l^−1^ glycerol in the production medium, 172 g l^−1^ crude glycerol was added. Additionally, 25 and 32.5 g l^−1^ NaCl in the production medium in the batch cultures with crude glycerol were obtained by adding 17.6 and 25.1 g l^−1^ NaCl, respectively. In the fed-batch cultures, the initial glycerol concentration was about 110 g l^−1^ and the initial volume of the culture broth was about 1.75 l. The total glycerol concentration of 250 g l^−1^ was obtained by adding two identical portions, i.e. 75 g l^−1^ glycerol, after 45 and 75 h of cultivation.

### Culture conditions

Growth was carried out in 0.3-l flasks containing 0.05 l growth medium on a rotary-shaker (CERTOMAT IS, Sartorius Stedim Biotech) at 29.5 °C at 140 rpm for 72 h. The shake-flasks experiment was conducted for 10 days in 0.3-l flasks containing 0.03 l of the appropriate medium under the same conditions as described above. Samples were taken at the end of the experiment. The batch cultivations were conducted in a 5-l stirred-tank reactor (BIOSTAT B-PLUS; Sartorius, Germany) with a working volume of 2.0 l at 29.5 °C. The aeration rate was fixed at 0.6 v v^−1^ min^−1^. The stirrer speed was adjusted to 800 rpm and the pH was maintained automatically at 3.0 by the addition of a 20 % (w v^−1^) NaOH solution. During the cultivations, samples were taken 2–3 times per day. All the cultures were cultivated until the complete consumption of carbon source. The shake-flasks experiment and all the batch cultures were performed in three and in two replications, respectively.

### Analysis of intracellular polyol content

Intracellular metabolites were analyzed in the biomass obtained at the end of bioreactor cultivations. After being washed twice with 0.05 M TrisHCl-buffer (pH 7.4), the biomass was treated with microwave radiation for 30 s. Extraction was performed with the use of 50 % ethanol at the boiling point for 1.5 h. The biomass-to-solvent ratio was 1:10. The mixture obtained was centrifuged (10 min; 4 °C; 5,500 rpm) and polyhydroxy alcohols in the supernatant were determined.

### Analytical methods

Samples from shake-flasks experiment and batch cultivations were centrifuged (10 min; 4 °C; 5,500 rpm). The biomass was determined gravimetrically after drying in a dryer at 105 °C. In the supernatants, the concentrations of glycerol, erythritol, mannitol, arabitol, ketoglutaric and citric acids were measured by HPLC using a Carbohydrate H+ Column (Thermo Scientific, Waltham, MA) coupled to a UV (λ = 210 nm) and refractive index (RI) detector (Shodex, http://www.shodex.com). The column was eluted with 25 mM trifluoroacetic acid (TFA) at 65 °C and a flow rate of 0.6 ml min^−1^.

The presence of arabitol in the supernatant was additionally confirmed after conversion into respective acetates using gas chromatography (GC) analysis. The supernatant sample was lyophilized in 203 K on a Christ Alpha 1-2 apparatus and the resulting powder was subjected to a reaction with acetic anhydride according to the method described earlier [36]. The acetates in the mixture obtained were identified by comparison of their retention times with those of standard acetates obtained from the commercially available alditols (glycerol, erythritol, rybitol, arabitol, mannitol, glucitol and galactitol) according to the same method. The structure of the synthesised acetates was confirmed by spectroscopic methods (IR, ^1^H NMR). All samples were analysed using GC on an Agilent 6890 N apparatus equipped with an FID detector using hydrogen as the carrier gas. The following conditions of analysis were applied: 70 % cyanopropyl polysilphenylene-siloxane column (TR FAME, 30 m × 0.25 mm× 0.25 μm), temperature programme: injector 150 °C, detector 250 °C, column 150 °C for 1 min, program rate 3 °C min^−1^, final temperature 260 °C for 5 min. The total time of analysis was 42.67 min.

### Statistical analysis

Data analysis was performed using Statistica 9.1 (StatSoft, Tulsa, OK).

## Results and discussion

### Shake-flasks experiment

In the shake-flasks experiment, nine *Y. lipolytica* yeast strains were examined for their ability to produce polyxydroxy alcohols such as erythritol, mannitol and arabitol from pure glycerol (100 g l^−1^) (Table 1). The pH of the buffered medium after 10 days of the experiment ranged from 2.5 to 2.8. The biomass concentration was the lowest (5.8 g l^−1^) for *Y. lipolytica* Wratislavia K1, whereas in the culture with *Y. lipolytica* CCY-29-26-3 it was almost twice higher and reached 10.5 g l^−1^. All strains under investigation produced erythritol with concentrations ranging from 20.0 to 35.5 g l^−1^, which corresponded to yields varying from 0.33 to 0.42 g g^−1^. The highest concentration of erythritol, observed in the culture with the A-15 strain of *Y. lipolytica*, was similar to the amount of this sugar alcohol obtained from glucose in the shake-flasks culture with the use of *Torula* sp. (35.2 g l^−1^) [10]. In the study conducted by Lee et al. [21] on erythritol biosynthesis from glucose by *Torula* sp., the addition of selected vitamins led to an increase in erythritol production from 46.3 to 58.3 g l^−1^. In another experiment, 60.3 g l^−1^ erythritol (corresponding to 0.25 g g^−1^ yield and volumetric productivity of 0.36 g l^−1^ h^−1^) was obtained when medium with a high glucose concentration (25 %) was applied [43]. In the shake-flasks experiment with *Trichosporonoides* sp. 331-1, yeast produced 43.0 and 37.4 g l^−1^ erythritol with a yield of 0.43 and 0.37 g g^−1^, respectively, when glucose and sucrose were used as substrates [4]. The effect of different carbon sources, including glycerol, on erythritol biosynthesis by *Pseudozyma tsukubaensis* was also investigated by Jeya et al. [12]. They found glycerol and lactose to be the worst substrates, yielding erythritol levels of 11.3 and 9.1 g l^−1^, respectively, whereas in the culture with glucose, 134 g l^−1^ erythritol was produced (which corresponded to a yield of 0.45 g g^−1^). The possibility of using *Y. lipolytica* yeast for erythritol biosynthesis was reported in a patent of the Mitsubishi Chemical Corporation Chiyoda-ku [47]. The patent focused on the use of glucose in concentrations of 10–60 %; however, the feasibility of erythritol biosynthesis from glycerol was also mentioned. In the shake flask culture with 200 g l^−1^ glycerol and *Y. lipolytica* ATCC 8661 strain, erythritol production of 43.2 g l^−1^ (corresponding to 0.21 g g^−1^ yield) was observed, thus the yield was significantly lower than in the presented work.

**Table 1:** Screening of *Yarrowia lipolytica* yeast strains to sugar alcohols biosynthesis from pure glycerol in a 10-day shake flask experiment

Strain	Biomass (g l^−1^)	Erythritol (g l^−1^)	Mannitol (g l^−1^)	Arabitol (g l^−1^)	*Y*_ER_ (g g^−1^)
CCY-29-26-3	10.5	22.97	2.63	2.33	0.33^a^
A-3	6.9	20.03	0.51	0.47	0.34^a^
Wratislavia 1.31	7.6	26.67	0.47	0.69	0.35^a^
A-2-4	8.4	22.47	0.48	1.09	0.35^ab^
A-1	7.5	27.03	0.2	0.3	0.36^ab^
CCY-29-26-4	8.1	26.7	0.96	2.21	0.36^ab^
A-UV’1	7.1	27.5	0.47	1.77	0.38^b^
A-15	9.3	35.53	1.07	1.72	0.42^c^
Wratislavia K1	5.8	23.23	<0.1	0.42	0.43^c^

Values with the same letters are not significantly different at the 0.05 level

In addition to erythritol, other polyols, such as mannitol, arabitol and rybitol, could be found in the culture broth during biosynthesis with yeast species of genera, e.g. *Debaryomyces*, *Hansenula*, *Pichia*, *Zygosaccharomyces* [27]. In our study, low amounts of mannitol (0.1–2.6 g l^−1^) and arabitol (0.3–2.3 g l^−1^) were detected in cultures with all strains after 10 days. Onishi and Suzuki [30] investigated the effect of different substrates on mannitol production by various yeasts in shake-flasks experiment. Of all the carbon sources examined, the most suitable for mannitol biosynthesis turned out to be glucose. Yeasts produced more than 44 g l^−1^ mannitol with a 0.30 g g^−1^ yield when glucose was added to the medium to a concentration of 20 % [14]. The results obtained showed that the use of different carbon sources at pH 6.5 in the culture with *Candida magnoliae* affected not only concentrations of polyols but, depending on its source, might also promote the production of particular compounds. In media with glucose, 7.2 g l^−1^ mannitol and 23.4 g l^−1^ erythritol were found, whereas 39 and 20 g l^−1^ mannitol and a lack of erythritol production were observed upon application of fructose and glycerol, respectively. Song et al. [44] isolated a strain of *C. magnoliae* that produced 67.0 g l^−1^ mannitol in fructose-containing medium.

Statistical analysis of the data (Table 1) indicated that the following strains of *Y. lipolytica* were the most suitable for sugar alcohol production: A-15, Wratislavia K1 and A-UV’1. Hence, these strains were used in further studies in batch cultures.

### Batch bioreactor cultures

In batch cultures, selected strains were examined for the production of erythritol and other polyols from pure glycerol and crude glycerol derived from biodiesel production, in both media not-supplemented and supplemented with NaCl to a total salt concentration of 2.5 and 3.25 % (Table 2). The parameters of erythritol production such as yield, volumetric productivity and specific production rate are presented in Table 3. The cultures were carried out until complete consumption of the carbon source in media, which lasted from 63 to 148 h depending on the strain used in the experiment, as well as the kind of glycerol and total salt concentration in the media. The addition of NaCl (2.5 %) to the media with pure glycerol caused a reduction in the biosynthesis time. This was also observed when crude glycerol was used, with the exception of the culture where strain A UV’1 was applied.

**Table 2:** Comparison of sugar alcohols production by *Y. lipolytica* strains on pure and crude glycerol media without and with NaCl supplementation

Strain	NaCl (%)	Time (h)	Biomass (g l^−1^)	Erythritol (g l^−1^)	Mannitol (g l^−1^)	Arabitol (g l^−1^)
Pure glycerol						
* Y. lipolytica* A UV’1	0	65.0	14.5	59.3	27.6	3.4
* Y. lipolytica* A UV’1	2.5	75.0	13.8	63.0	8.8	9.2
* Y. lipolytica* A-15	0	95.0	13.8	28.0	23.0	1.3
* Y. lipolytica* A-15	2.5	75.0	15.8	71.0	8.0	1.8
* Y. lipolytica* Wratislavia K1	0	99.0	14.9	42.0	12.8	4.3
* Y. lipolytica* Wratislavia K1	2.5	97.5	13.7	84.1	3.8	0.5
Crude glycerol						
* Y. lipolytica* A UV’1	n.s.	74.5	16.4	58.9	13.7	5.6
* Y. lipolytica* A UV’1	2.5	63.0	16.5	51.0	5.7	4.6
* Y. lipolytica* A UV’1	3.25	148.0	17.9	40.0	9.0	4.9
* Y. lipolytica* A-15	n.s.	119.0	13.5	35.0	13.2	3.5
* Y. lipolytica* A-15	2.5	90.0	15.8	64.9	14.9	2.2
* Y. lipolytica* A-15	3.25	94.5	15.9	65.0	5.3	1.2
* Y. lipolytica* Wratislavia K1	n.s	105.0	18.5	70.0	10.0	0.9
* Y. lipolytica* Wratislavia K1	2.5	80.0	17.2	80.0	4.5	n.d.
* Y. lipolytica* Wratislavia K1	3.25	127.0	15.8	80.5	2.6	0.3

*n.s.* not supplemented, *n.d*. not detected

**Table 3:** Parameters of erythritol and mannitol biosynthesis by *Y. lipolytica* strains on pure and crude glycerol media without and with NaCl addition

Strain	NaCl (%)	Erythritol			Mannitol		
		*Y*_ER_ (g g^−1^)	*Q*_ER_ (g l^−1^ h^−1^)	*q*_ER_ (g g^−1^ h^−1^)	*Y*_M_ (g g^−1^)	*Q*_M_ (g l^−1^ h^−1^)	*q*_M_ (g g^−1^ h^−1^)
Pure glycerol							
* Y. lipolytica* A UV’1	0	0.35	0.91	0.063	0.16	0.42	0.029
* Y. lipolytica* A UV’1	2.5	0.39	0.84	0.061	0.05	0.12	0.009
* Y. lipolytica* A-15	0	0.19	0.29	0.021	0.16	0.24	0.018
* Y. lipolytica* A-15	2.5	0.44	0.95	0.060	0.05	0.11	0.007
* Y. lipolytica* Wratislavia K1	0	0.22	0.35	0.022	0.07	0.12	0.007
* Y. lipolytica* Wratislavia K1	2.5	0.50	0.86	0.063	0.02	0.04	0.003
Crude glycerol							
* Y. lipolytica* A UV’1	n.s.	0.38	0.79	0.048	0.09	0.22	0.014
* Y. lipolytica* A UV’1	2.5	0.34	0.81	0.049	0.04	0.09	0.005
* Y. lipolytica* A UV’1	3.25	0.28	0.27	0.015	0.06	0.06	0.003
* Y. lipolytica* A-15	n.s.	0.23	0.29	0.022	0.09	0.11	0.008
* Y. lipolytica* A-15	2.5	0.39	0.72	0.046	0.09	0.17	0.010
* Y. lipolytica* A-15	3.25	0.39	0.69	0.043	0.03	0.06	0.004
* Y. lipolytica* Wratislavia K1	n.s.	0.44	0.67	0.036	0.06	0.10	0.005
* Y. lipolytica* Wratislavia K1	2.5	0.49	1.00	0.058	0.03	0.06	0.003
* Y. lipolytica* Wratislavia K1	3.25	0.49	0.63	0.039	0.02	0.02	0.002

*n.s.* not supplemented

It is known that impurities present in raw waste substrates can have a major impact on the growth of microorganisms and their metabolic processes. In our study, the biomass concentration ranged from 13.8 to 15.8 g l^−1^ when pure glycerol was used (Table 2). In cultures with crude glycerol, the biomass concentration of Wratislavia K1 and A UV’1 strains was higher than that of cultures with a pure substrate, which suggests that the cells of these strains utilized the impurities contained in the crude glycerol. The use of a crude substrate did not affect biomass production by the A-15 strain.

Erythritol has been produced commercially from glucose, derived from chemical or enzymatic hydrolysis of wheat and corn starch, using *Aureobasidium* sp. and *Pseudozyma tsukubaensis*. Other erythritol-producing microorganisms include osmophilic yeasts and yeast-like fungi such as *C. magnoliae*, *Moniliella tomentosa*, *Torula* sp., *Trichosporon* sp. and *Trichosporonoides* sp. [27]. The level of erythritol obtained from glucose during batch cultures depended on the microorganism and the composition of the substrate applied in the experiments and ranged widely from 18 to 243 g l^−1^ [12, 15, 18, 21, 24, 25, 29, 33, 38, 50]. Generally, for these microorganisms it was observed that a high initial glucose concentration improved erythritol production. Previous studies showed that *Y. lipolytica* yeast were able to produce erythritol using glycerol as the sole carbon and energy source at low pH values (3.0) of media whereas glucose was found to be a worse substrate [37].

In this study, generally more erythritol was produced in cultures with crude glycerol than those with pure substrate; however, the addition of NaCl to the medium resulted in an upsurge of erythritol biosynthesis. As shown in Table 2, the highest amount of erythritol (84.1 g l^−1^) was produced by strain Wratislavia K1 in the culture with pure glycerol with 2.5 % of NaCl. For this strain, erythritol production yield increased significantly (from 0.22 to 0.50 g g^−1^) when salt was added to media containing pure glycerol (Table 3). The result was similar to the yield (0.49 g g^−1^) observed by Lee et al. [20] during erythritol biosynthesis from glucose by *Torula* sp. The highest reported yield of erythritol production was obtained on glucose medium with the 440 N61188-12 strain of *Moniliella* sp. and reached 0.63 g g^−1^ [25].

Compared to the cultures with pure glycerol only, a notable increase in yield (2x) was observed for the Wratislavia K1 strain when crude glycerol was used (Table 3). This may be explained by the influence of the original components of the raw substrate, which contained NaCl (see “Materials and methods”), among many other impurities. Studies on polyols production have shown that the use of microorganisms with a relatively strong tolerance for a high concentration of salts and sugars resulted in higher biosynthesis yields [26]. According to Kim et al. [16], the production of erythritol by *Torula* sp. was maximal at 0.3 M NaCl or 0.4 M KCl in the culture broth, while cell growth and glucose consumption rates decreased as salt concentrations increased over 0.3 M. In this study, the highest volumetric erythritol production rate (1.0 g l^−1^ h^−1^) was obtained for Wratislavia K1 strain in a culture with crude glycerol and NaCl supplementation to 2.5 % (Table 3). It is worth noting that the higher concentration of salt (3.25 %) caused a decrease in volumetric productivity. The volumetric production rates obtained in this work using strains of *Y. lipolytica* are significantly higher than those obtained when *Trigonopsis variabilis* and *Moniliella* sp. were used for erythritol biosynthesis [15, 24]. In comparison to the work presented here, slightly higher volumetric erythritol productivity (1.08 g l^−1^ h^−1^) was demonstrated in medium with 14 % glucose, whereas the value increased to 1.23 g l^−1^ h^−1^ when 30 % glucose was applied [33]. In erythritol biosynthesis with *P. tsukubaensis* [12] and *Moniliella* sp. N61188-12 [25], volumetric productivity reached 1.65 and 1.98 g l^−1^ h^−1^, respectively.

Generally, the addition of NaCl to the medium resulted in a higher specific rate of erythritol formation (Table 3). The highest values of this parameter (0.063 g g^−1^ h^−1^) were obtained on pure glycerol medium, although without NaCl in biosynthesis by strain A UV’1 and with a salt addition when Wratislavia K1 was used. The use of other microorganisms and glucose as a substrate resulted in varied values of this parameter, ranging between 0.045 and 0.098 g g^−1^ h^−1^ [18, 29, 33].

It is worth noting that, in cultures with pure glycerol without NaCl, a significant amount of mannitol was observed, especially for A UV’1 and A-15 strains (Table 2). The A UV’1 strain produced 27.6 g l^−1^ mannitol, which corresponded to 0.16 g g^−1^ yield and a productivity of 0.42 g l^−1^ h^−1^ (Table 3). A lower concentration of mannitol (23.0 g l^−1^) was obtained with the A-15 strain but its amount was comparable to that of erythritol produced by this strain. After addition of salt to the medium, three-fold lower mannitol production was observed. Salt addition to media with crude glycerol caused similar effects on erythritol biosynthesis as observed with pure glycerol—production of erythritol doubled and the inhibition of mannitol production was not as significant as before. However, in biosynthesis on crude glycerol with strain A UV’1, NaCl supplementation resulted in a decreased erythritol concentration from 58.9 g l^−1^ without salt to 40.0 g l^−1^ with 3.25 % NaCl, whereas the erythritol-to-mannitol ratio remained unchanged (4:1). It is clear that erythritol and mannitol biosynthesis was dependent on the substrate used, as in crude glycerol a higher concentration of erythritol and lower concentration of mannitol were obtained, which was probably associated with the presence of salt in this substrate. The inhibitory effect of salt on mannitol production observed in this study is consistent with the results presented by Onishi and Suzuki [30]. The conversion of erythritol- to mannitol-production by *C. zeylanoides* was investigated by Hattori and Suzuki [11], but the factor affecting the erythritol-to-mannitol ratio was the phosphate source and its concentration in the medium.

Fructose and glucose-containing media have been found to be the most suitable for the production of mannitol. Mannitol can be produced by several microorganisms, including yeasts such as *C. magnoliae*, *C. zeylanoides*, the fungi *Aspergillus* and bacteria, especially lactic acid bacteria [42, 45]. The production of mannitol with *C. magnoliae* HH-01 yeast reached 223 g l^−1^ when fructose-glucose medium was supplemented with Ca^++^ and Cu^++^ [23].

Mannitol and erythritol were present in the culture broth as by-products during citric acid biosynthesis from glycerol at pH 5.5 by various strains of *Y. lipolytica*. Worthy of note is that, after a significant reduction in glycerol concentration, polyols were generally utilized, which extended the duration of citric acid synthesis. The mannitol concentration at the end of citric acid biosynthesis did not exceed 8.3 g l^−1^ [40]. In the literature, there is little information about the production of mannitol by *Y. lipolytica* [2, 8]. It is interesting that mannitol, at concentrations of 5.1 and 6.0 g l^−1^, was the principal extra-cellular metabolite and the only polyol produced in nitrogen-limited culture conditions at pH 4.5 by strains LFMB 19 and LFMB 20, respectively [2]. According to Chatzifragkou et al. [8], mannitol production by *Y. lipolytica* LFMB strain 19 was enhanced by increasing the glycerol concentration. For this strain, at a high initial glycerol concentration (90 g l^−1^), re-consumption of metabolic products was not noted and the final mannitol concentration was 19.4 g l^−1^.

In this study, the parameters of mannitol biosynthesis, i.e. yield, volumetric and specific production rate, were generally lower than the values obtained for erythritol production (Table 3). As mentioned above, the best results were observed for the A UV’1 strain; however, the results are lower than literature findings. Lee et al. [23] reported that, in a culture with *C. magnoliae* HH-01 in fructose medium, the mannitol production yield reached 0.79 g g^−1^, whereas the volumetric production rate was 1.72 g l^−1^ h^−1^.

In the current study, various amounts of arabitol, citric and α-ketoglutaric acids were found in the culture broth. In all cultures, the amounts of these acids were relatively low. The concentration of citric and α-ketoglutaric acid did not exceed 4.4 and 3.1 g l^−1^, respectively (data not shown). Table 2 shows that the highest concentration of arabitol (9.2 g l^−1^) was obtained upon the use of the A UV’1 strain. It is important to note that for this strain, when salt was present in the pure glycerol medium, a notably higher concentration of arabitol was produced. It is possible that, for this strain, arabitol is an important compound in counteracting osmotic stress. It is also worth noting that the literature lacks information about the possibility of arabitol production using *Y. lipolytica*. Koganti et al. [17] reported on using glycerol as a carbon source for arabitol biosynthesis with *D. hansenii*.

### Intracellular polyols accumulation

Salinity is one of the main stress factors affecting the growth of microorganisms [6]. When exposed to osmotic stress, microorganisms induce many molecular, biochemical and physiological changes, known as the “osmotic stress response” [7]. The production and intracellular accumulation of low molecular mass compounds plays a major role in counteracting the outflow of water and maintaining the cytoplasmic water activity at an equal level with the surrounding environment [19, 48]. Osmophilic microorganisms may accumulate one or more compatible solutes [28]. In yeast, the most pronounced response is enhanced intracellular accumulation of polyhydroxy alcohols such as glycerol, arabitol, mannitol and erythritol; however, these polyols are also produced as an integral part of normal yeast growth [13, 26]. Polyols accumulation is dependent on yeast species, the phase of growth, and the carbon source [48].

**Fig. 1:**
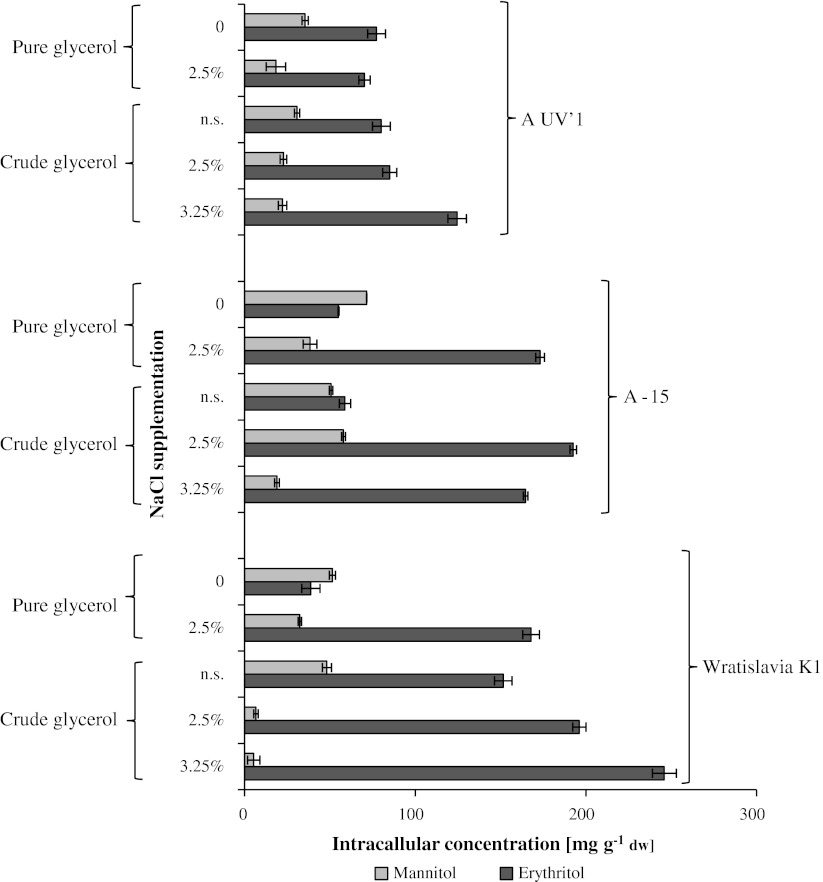
Intracellular concentration of polyhydroxy alcohols in *Yarrowia lipolytica* strains after batch cultivation in glycerol media with and without (n.s.) NaCl supplementation. Each *point* is the mean value of two independent cultures

In this research, remarkable amounts of erythritol and mannitol were found in the yeast biomass (Fig. 1). The intracellular concentration of erythritol increased when NaCl was added to the medium. The highest erythritol concentration (245.9 mg g^−1^ dw) was found in the cells of Wratislavia K1 harvested after the culture with crude glycerol supplemented with 3.25 % NaCl, whereas the lowest concentration of 38.8 mg g^−1^ dw was obtained after the culture with the same strain in pure glycerol media without salt addition. In the medium without salt supplementation, a higher level of intracellular erythritol was obtained when crude glycerol was used, which can be explained by the induction of osmotic adjustment caused by the salt originally contained in this raw substrate. In the non-stressed cells (media with pure glycerol and without salt addition), the intracellular concentration of mannitol varied from 35.4 to 71.5 mg g^−1^ dw and was higher than that of erythritol, with the exception of A UV’1 strain. In contrast to erythritol, the intracellular mannitol concentration in the cultures with crude glycerol was lower than when pure substrate was applied. When NaCl was added to media with both pure and crude glycerol, the intracellular content of mannitol decreased; however, the ratio of its intracellular-to-extracellular concentration generally increased. This finding suggests that mannitol also plays an important role in counteracting osmotic stress in the cells of investigated strains of *Y. lipolytica*. There is a lack of information in the literature about intracellular concentration of polyols in the cells of *Y. lipolytica*. In *C. magnoliae,* lower intracellular concentrations of mannitol (>1 g l^−1^) were obtained when NaCl was added in comparison to amounts obtained on glucose, irrespective of water activity [48]. The cells of *Pichia sorbitophila* after 5 min exposure to salt stress (0.86 M NaCl) accumulated 32.13 mg g^−1^ dw erythritol—which was twice as much in comparison to non-stressed cells [13]. Halotolerant black yeast, known for their ability to grow even in concentrations of 5.2 M NaCl, accumulated 1.95–88.8 mg g^−1^ dw erythritol [34]. Lee et al. [20] reported that *Torula* sp. in media with glucose and minerals (Mn^+^ or Cu^+^) was able to accumulate 0.9–1.5 g l^−1^ of intracellular erythritol. Cells of *Candida sake* were able to accumulate up to 1.25 mg g^−1^ fw arabitol and less than 0.9 mg g^−1^ fw erythritol and mannitol when grown in dextrose media with glycerol [46].

### Fed-batch cultures

**Fig. 2:**
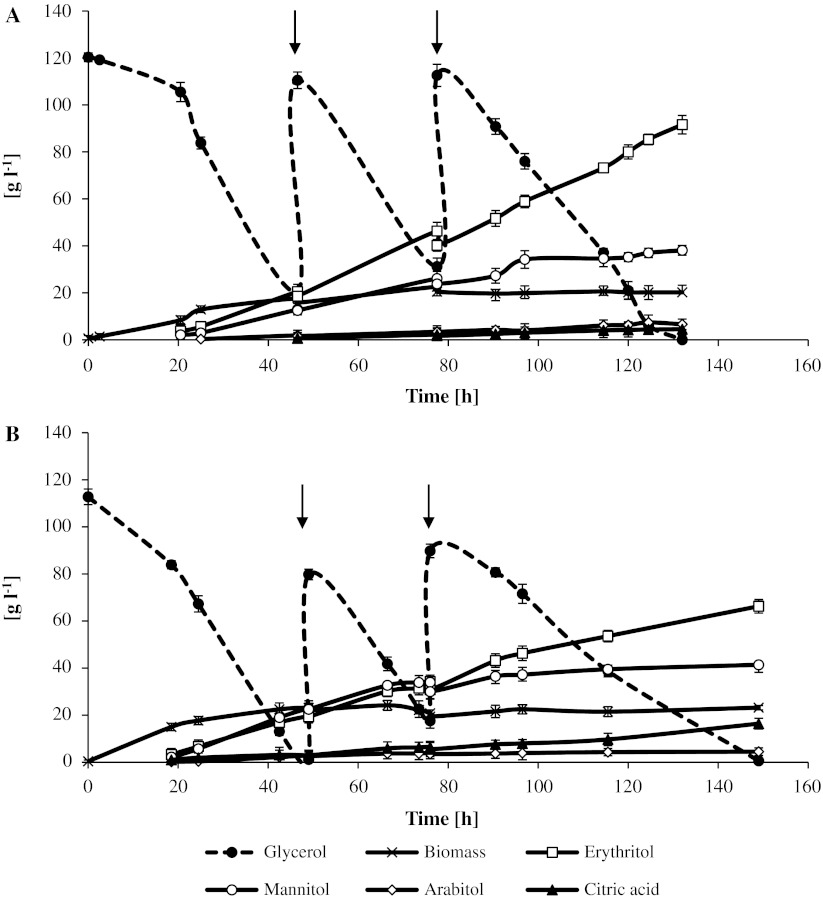
Time profiles of biomass, glycerol, sugar alcohols and citric acid for the *Y. lipolytica* A UV’1 (**a**) and A-15 (**b**) during fed-batch cultures on glycerol. Glycerol (98 %) was periodically fed into the fermentor as indicated by the *arrows*. Data from duplicate fermentations are shown

The simultaneous production of erythritol and mannitol in fed-batch cultures by *Y. lipolytica* A UV’1 and A-15 was investigated using pure glycerol medium without NaCl addition. Glycerol was added into the culture broth in two portions when its concentration fell below 3 % (Fig. 2).

The biomass concentration of both strains was higher than in batch cultures, as the initial volume in fed-batch system was lower (1.75 l) and increased to a final 2 l after the addition of glycerol. The erythritol concentration obtained in the fed-batch cultures reached 91.6 and 66.3 g l^−1^, with A UV’1 and A-15 strain, respectively. For both strains, the fed-batch cultures resulted in a slight increase of the erythritol conversion yield in comparison to the batch cultures (Table 4). Rymowicz et al. [37] reported that using crude glycerol (300 g l^−1^) in a fed-batch system with *Y. lipolytica* Wratislavia K1 resulted in a higher concentration of erythritol (170 g l^−1^) with a 0.56 g g^−1^ conversion yield and erythritol productivity of 1.0 g l^−1^ h^−1^, whereas only 23 g l^−1^ of erythritol with 0.14 g g^−1^ yield was produced in glucose medium. In comparison to the present study, comparable results in fed-batch mode were achieved by Savergave et al. [43] in glucose medium with *C. magnoliae* R23. The mutant R23 produced 87.8 g l^−1^ erythritol corresponding to 0.31 g g^−1^ erythritol yield. In other investigations, the application of glucose media (35–40 %) and different yeast genus/species resulted in a concentration of erythritol varying from 187 to 245 g l^−1^ with volumetric productivity ranging from 0.76 to 2.89 g l^−1^ h^−1^ [12, 25, 29, 38, 43].

**Table 4:** Parameters of fed-batch erythritol and mannitol biosynthesis by *Y. lipolytica* strains on pure glycerol without NaCl supplementation

Strain	Erythritol			Mannitol		
	*Y*_ER_ (g g^−1^)	*Q*_ER_ (g l^−1^ h^−1^)	*q*_ER_ (g g^−1^ h^−1^)	*Y*_M_ (g g^−1^)	*Q*_M_ (g l^−1^ h^−1^)	*q*_M_ (g g^−1^ h^−1^)
*Y. lipolytica* A UV’1	0.37	0.69	0.034	0.15	0.29	0.014
*Y. lipolytica* A-15	0.27	0.44	0.020	0.17	0.28	0.013

As illustrated in Fig. 2, the mannitol concentration at the end of the process for both strains was similar, reaching 38.1 and 41.4 g l^−1^ in the culture with A UV’1 and A-15, respectively. In the case of Wratislavia K1, the mannitol concentration was obtained 12 and 23 g l^−1^ in the glycerol and glucose media, respectively [37]. In the fed-batch cultures with optimized fructose media, different strains of *C. magnoliae* produced about 211 g l^−1^ mannitol, which corresponded to ~0.84 g g^−1^ yield [22, 44].

In this study, the wild-type strain A-15 produced noticeable amounts of citric acids (16.4 g l^−1^) in the fed-batch system (Fig. 2a), which was not observed in the batch culture process. This is interesting because the production of citric acid by *Y. lipolytica* yeast at low pH (3.0) is generally very low [37]. The presence of citric acid might have been caused by a high nitrogen deficiency due to the high biomass concentration. Further studies are necessary to eliminate this by-product and enhance mannitol production.

## Conclusions

While erythritol and mannitol production by yeasts has been known for many years, their production with the use of glycerol as a substrate has rarely been the core focus of published reports. The results obtained in the presented work confirm that the application of *Y. lipolytica* yeast has considerable potential for both erythritol and mannitol biosynthesis. Additional advantage of the process is a low pH value (3.0) during biosynthesis, which protects the culture against bacterial contamination and thereby supports the development of continuous fermentation procedures. The other benefit of the process is that it needs only a simple cultivation medium containing inorganic salts and glycerol as a carbon source. Furthermore, biodiesel-derived crude glycerol was demonstrated to be an adequate substrate for this process. Thus, the presented results provide an alternative means of this by-product valorization to value-added products, i.e. sugar alcohols.

The highest-yielding strain, *Y. lipolytica* Wratislavia K1, after 4 days produced 84.1 and 80.0 g l^−1^ erythritol corresponding to a yield of 0.5 and 0.49 g g^−1^, respectively, from pure and crude glycerol in the medium supplemented with NaCl (to 2.5 %). The total concentration of by-products (other polyols and organic acids) did not exceed 8 g l^−1^. This work has also identified two strains (A UV’1 and A-15) useful for the simultaneous biosynthesis of erythritol and mannitol. In the fed-batch process, the A UV’1 strain produced 91.6 g l^−1^ erythritol and 38.1 g l^−1^ mannitol in pure glycerol media without salt supplementation. In addition, this study demonstrated that the erythritol-to-mannitol ratio may be changed by using NaCl. The results achieved indicate that the addition of NaCl enhances erythritol production and, simultaneously, inhibits mannitol formation.

## Abbreviations

*Y*_ER_, *Y*_M_: Production yield with respect to substrate consumed (*ER* erythritol,* M* mannitol)
*Q*_ER_, *Q*_M_: Volumetric productivity
*q*_ER_, *q*_M_: Specific production rate
